# Basal ganglia vulnerability to oxidative stress

**DOI:** 10.3389/fnins.2014.00080

**Published:** 2014-04-21

**Authors:** Nina Jensen, João R. M. Oliveira

**Affiliations:** ^1^Department of Clinical Medicine, Aarhus UniversityAarhus, Denmark; ^2^Department of Molecular Biology and Genetics, Aarhus UniversityAarhus, Denmark; ^3^Keizo Asami Laboratory, Department of Neuropsychiatry, Federal University of PernambucoRecife, Pernambuco, Brazil

**Keywords:** neurodegeneration, essential fatty acids, oxidative stress, DHA, resilience

Increased levels of oxidative stress (OS) in the brain have been linked to the etiology of several neurodegenerative diseases, e.g., Alzheimer's and Parkinson's disease (Navarro and Boveris, 2009; Melo et al., 2011; Radak et al., 2011; Sultana et al., 2013). A possible cause of increased OS levels is an imbalance in the omega-6 (n-6) and omega-3 (n-3) polyunsaturated fatty acids (PUFAs) (Kiecolt-Glaser et al., 2013). The main PUFA in a westernized diet is the essential fatty acid (EFA) linoleic acid (n-6), a major component of plant oils, followed by the EFA α-linoleic acid (n-3).

EFA deprived rats over two generations display reduced numbers and size of dopaminergic neurons in the substantia nigra (SN) (Passos et al., 2012). To evaluate this finding, Cardoso et al. (2012) studied the effect of EFA deprivation on OS levels and neurodegeneration. Rats were depleted of both linoleic and α-linoleic acid over two generations. First (F1) and second (F2) generations were studied at 90–110 and 30–42 days of age, respectively. A decrease in docosahexaenoic acid (DHA) (n-3) in midbrain phospholipids was detected in both F1 and F2 animals (approximately 28 and 50%, respectively). Conversely, docosapentaenoic acid (DPA, n-6) was increased, which is likely to change membrane fluidity, due to one less double bond (Eldho et al., 2003). No change was found in Arachinoid acid (AA) (n-6) levels in either group relative to controls. Thus the n-6/n-3 ratio was increased in the EFA depleted rats.

OS was estimated by assessing lipid peroxidation (LP) using the thiobarbituric acid reaction method (TBARS). No LP in either SN or corpus striatum (CS) in the F1 rats was found, correlating with an increase in total superoxide dismutase activity. In F2 rats a decrease in catalase activity, an increase in LP, and degeneration of dopaminergic and non-dopaminergic neurons in SN was observed. The CS showed increased total superoxide dismutase activity but no increase in LP or neuron damage, suggesting a greater resilience to EFA deprivation than the SN. However, in a follow up study the striatum of EFA depleted rats (F2) also showed affects at 90–110 days of age (Cardoso et al., 2013). These results indicate that CS resilience to OS is only present in the young animals.

A western diet typically contains more n-6 PUFAs than n-3 PUFAs, which is problematic due to the adverse function of these PUFAs and their metabolites. Linoleic acid and α-linoleic acid are metabolized to AA and eicosapentaenoic acid (EPA) (n-3). AA and EPA are metabolized to DPA and DHA, respectively, or to eicosanoids. The eicosanoids derived from AA are mostly pro-inflammatory, whereas EPA derived eicosanoids are predominantly anti-inflammatory (Schmitz and Ecker, 2008). In addition, the n-6/n-3 ratio is important for several functions, including neuron membrane fluidity. AA, DHA, and other PUFAs are incorporated into the phospholipids of membranes, where their effect on membrane fluidity affects the functions of membrane transporters, channels and receptors (Yehuda et al., 2002; Schmitz and Ecker, 2008). Decreased DHA in particular can be harmful and is linked to OS and neurodegeneration (Malcolm et al., 1998; Yavin et al., 2002; Schmitz and Ecker, 2008; Jansen and Kliaan, 2014). DHA has been proposed as a treatment for some neurodegenerative disorders including Parkinson's and Alzheimer's disease (Jansen and Kliaan, 2014). Studies show varying effects of n-3 fatty acid supplementation on cognitive abilities in animal models and humans (Luchtman and Song, 2013).

This article discusses how an imbalance in EFAs might be linked to neurodegenerative diseases through increased OS, and that this effect is cumulative over generations. However, as F1 and F2 animals are studied at different ages, comparison of the two generations is difficult. The authors do not explain why these ages were chosen, but one can argue that as OS is often increased in old age, the fact that the findings in the younger F2 animals are not present in the older F1 animals, actually strengthens these findings.

The SN is less resilient to OS than other brain regions (Kidd, 2000), which might aid in explaining why Parkinson's disease, characterized by death of dopaminergic neurons in the SN, is among the commonest neurodegenerative diseases. Intriguingly, markers of adult neurogenesis have recently been found in the striatum, as well as in the caudate nucleus and putamen, but not in the cerebellum, or cerebral cortex (Tong et al., 2011; Ernst et al., 2014). This might play a role in differential resilience to OS and other insults, in addition to the differences in defensive mechanisms to OS. Cardoso et al. reported a link between DHA depletion in phospholipids and increased OS in the form of LP, as well as a differential response in SN and CS. If a recovery study, where EFAs are returned to deprived F2 animals, could reverse the findings, it would strengthen this link between DHA depletion and OS.

The work by Cardoso et al. contributes to the understanding of differential brain resilience to insults such as increased levels of OS. A better understanding of the function of DHA and its possible role in neuroprotection, as well as the mechanisms that lead to EFA deprivation mediated OS will be crucial to discern the etiology of several neurodegenerative diseases and in the development of future treatments.

## Conflict of interest statement

The authors declare that the research was conducted in the absence of any commercial or financial relationships that could be construed as a potential conflict of interest.
